# Bilingual advantages in executive functioning: problems in convergent validity, discriminant validity, and the identification of the theoretical constructs

**DOI:** 10.3389/fpsyg.2014.00962

**Published:** 2014-09-09

**Authors:** Kenneth R. Paap, Oliver Sawi

**Affiliations:** ^1^Language Attention and Cognitive Engineering Lab, Department of Psychology, San Francisco State UniversitySan Francisco, CA, USA; ^2^Department of Psychology, University of ConnecticutStorrs, CT, USA

**Keywords:** executive processing, reliability, validity, antisaccade, flanker, Simon, switching, bilingualism

## Abstract

A sample of 58 bilingual and 62 monolingual university students completed four tasks commonly used to test for bilingual advantages in executive functioning (EF): antisaccade, attentional network test, Simon, and color-shape switching. Across the four tasks, 13 different indices were derived that are assumed to reflect individual differences in inhibitory control, monitoring, or switching. The effects of bilingualism on the 13 measures were explored by directly comparing the means of the two language groups and through regression analyses using a continuous measure of bilingualism and multiple demographic characteristics as predictors. Across the 13 different measures and two types of data analysis there were very few significant results and those that did occur supported a monolingual advantage. An equally important goal was to assess the convergent validity through cross-task correlations of indices assume to measure the same component of executive functioning. Most of the correlations using difference-score measures were non-significant and many near zero. Although modestly higher levels of convergent validity are sometimes reported, a review of the existing literature suggests that bilingual advantages (or disadvantages) may reflect task-specific differences that are unlikely to generalize to important general differences in EF. Finally, as cautioned by Salthouse, assumed measures of executive functioning may also be threatened by a lack of discriminant validity that separates individual or group differences in EF from those in general fluid intelligence or simple processing speed.

## Introduction

Executive functions (EFs) consist of a set of general-purpose control processes believed to be central to the self-regulation of thoughts and behaviors that are instrumental to accomplishing goals. Across many theoretical frameworks these functions include planning, organizing, sequencing, problem solving, decision-making, goal selection, switching between task sets, monitoring for conflict, monitoring for task-relevant information, monitoring performance levels, updating working memory, interference suppression, and inhibiting prepotent responses. The functions assigned to EF are quite broad, many appear to be related to thinking in general, and this has led Salthouse (2005) and others to consider if the concept of EF is different from that of general fluid intelligence (gF). This concern will be examined in a discussion of discriminant validity.

From a neuropsychological perspective the construct of EF is often viewed as a set of interrelated component processes all involving the prefrontal cortex (PFC) with each component recruiting additional areas of cortical function. This componential framework allows for the possibility that the related components have some degree of anatomical and functional independence. Thus, individuals may vary in terms of overall EF ability or with respect to specific components^1^. If EFs are general-purpose then individuals who excel in, say, a measure of inhibitory control in one task should also show little interference (excellent inhibitory control) in a different task. That is, indices obtained in different tasks, but assumed to measure the same component of EF, should correlate and show convergent validity. One important purpose of the present study is to assess the convergent validity of 13 measures of EF obtained in the antisaccade, attentional network test (ANT)^2^, Simon, and color-shape switching task. These four tasks were selected because they have dominated the non-verbal tests for bilingual advantages in EF, particularly for samples of young adults and the elderly.

## Unity and diversity of EF

**Figure 1:**
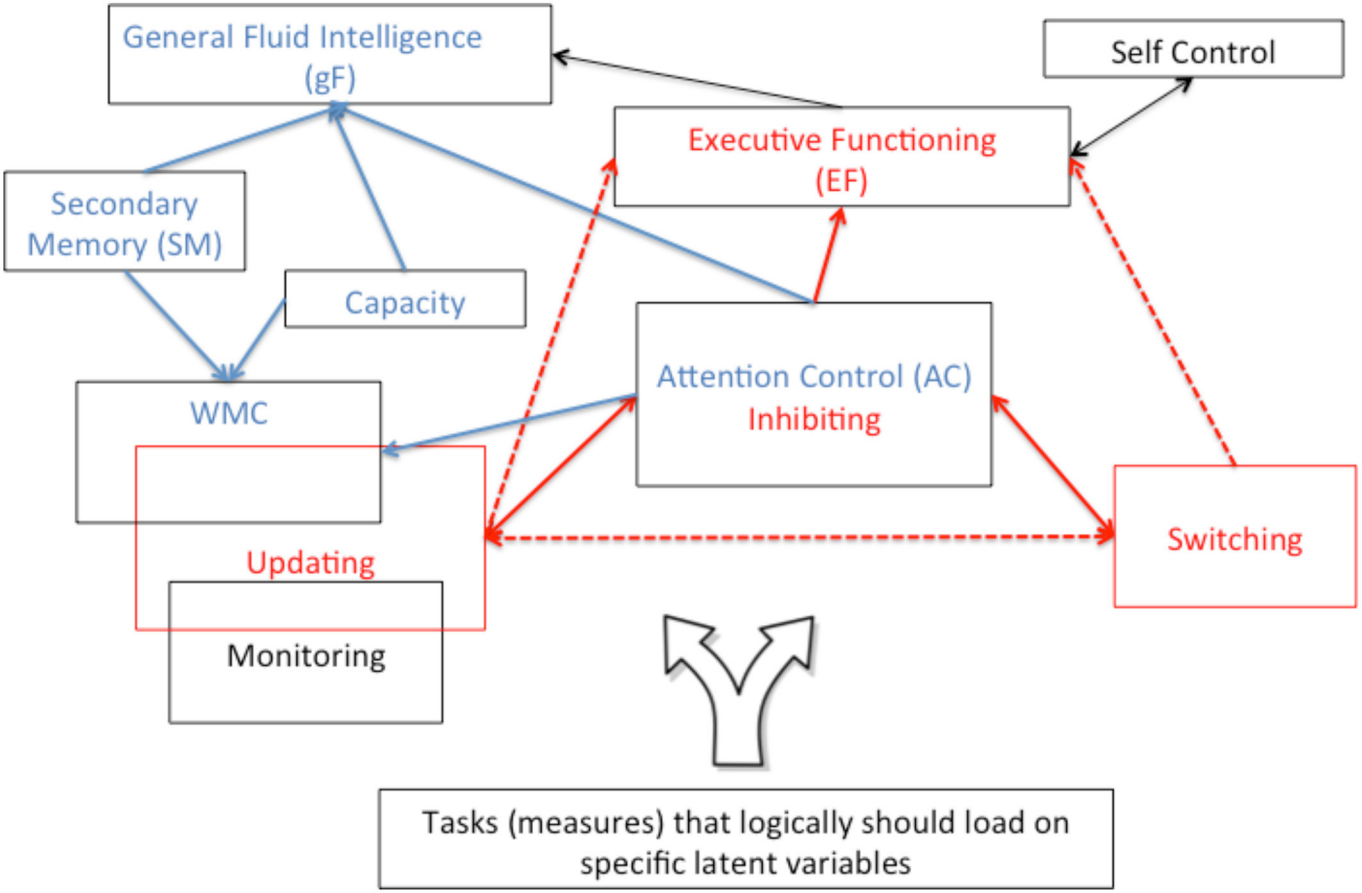
**A hierarchical schema with performance on specific tasks at the bottom and higher cognitive abilities (e.g., general fluid intelligence) at the top**. The Miyake and Friedman (2012) unity and diversity model of executive functioning is presented in red; whereas the Unsworth et al. (2014) multifacet model of working memory is represented in blue.

The influential work of Miyake and Friedman (Miyake et al., 2000; Friedman and Miyake, 2004; Friedman et al., 2008; and Miyake and Friedman, 2012) shows evidence for three components of EF: updating, switching^3^, and inhibition. Confirmatory factor analyses (CFA) were based on measures from three different tasks for each of the three hypothesized components. For each latent variable (viz., updating, switching, inhibition) the three observed variables linked to the same latent variable, are correlated with one another, and result in standardized factor loadings ranging from 0.40 to 0.71. At the higher level the three latent variables correlate with one another and this is consistent with the assumption that each contributes to a common EF. When the same data are reanalyzed with a second order CFA where the three latent variables are nested under a common EF latent variable, the nine observed measures all load on common EF with two of the components (updating and shifting) still making unique contributions. These findings support the assumption of a general EF ability with separable updating and switching components and an inhibition component that is not separable and that is weakly to moderately linked to the common EF ability. Because the best models of the data include both common and componential levels Miyake and Friedman propose that EF has both *unity* and *diversity*. The Miyake and Friedman model is represented in red in Figure 1 with solid and broken lines representing stronger and weaker associations, respectively.

## Bilingual advantages in EF

In the last decade there have been numerous reports of bilinguals out performing monolinguals on a variety of tasks assumed to reflect EF. These results have led to a widely held belief that most bilinguals enjoy an advantage over monolinguals in EF. In a recent review Bialystok (2011) stated that “Studies have shown that bilingual individuals *consistently* [emphasis added] outperform their monolingual counterparts on tasks involving executive control” p. 229. In a follow-up it is reported that “… bilinguals *at all ages* [emphasis added] demonstrate better executive control than monolinguals matched in age and other background factors” (Bialystok et al., 2012, p. 212). Similarly, Kroll and Bialystok (2013) observed that “… studies of executive function demonstrate a bilingual advantage, with bilinguals outperforming their monolingual counterparts on tasks that required *ignoring irrelevant information*, *task switching*, and *resolving conflict* [emphasis added]” (p. 2). These unqualified conclusions are likely to lead to inferences that benefits accrue from most types of bilingual experiences and that they transfer to general abilities across both verbal and non-verbal domains. Closer inspection of the full range of outcomes suggests that greater caution be exercised, as there are a growing number of failures to find differences between bilinguals and monolinguals. Furthermore, when differences are found the psychometric properties of the measures frequently do not support generalizing the performance advantage from the specific laboratory task(s) employed to domain-general and real-world scenarios.

Hilchey and Klein (2011) reviewed 31 experiments using non-verbal interference tasks (e.g., Simon or flanker tasks) and concluded that evidence for a bilingual advantage in inhibitory control in both children and young adults is rare and that the collective evidence “… is simply inconsistent with the proposal that bilingualism has a general positive effect on inhibitory control processes” (p. 629). In contrast, Hilchey and Klein were impressed with the relative frequency of bilingual advantages in measures of monitoring, but in an update of their 2011 review Hilchey et al. (2014) observe that the influx of new data strongly repudiates their earlier conclusion that managing two languages leads to bilingual advantages in monitoring.

### Bilingual advantages and small sample sizes

**Figure 2:**
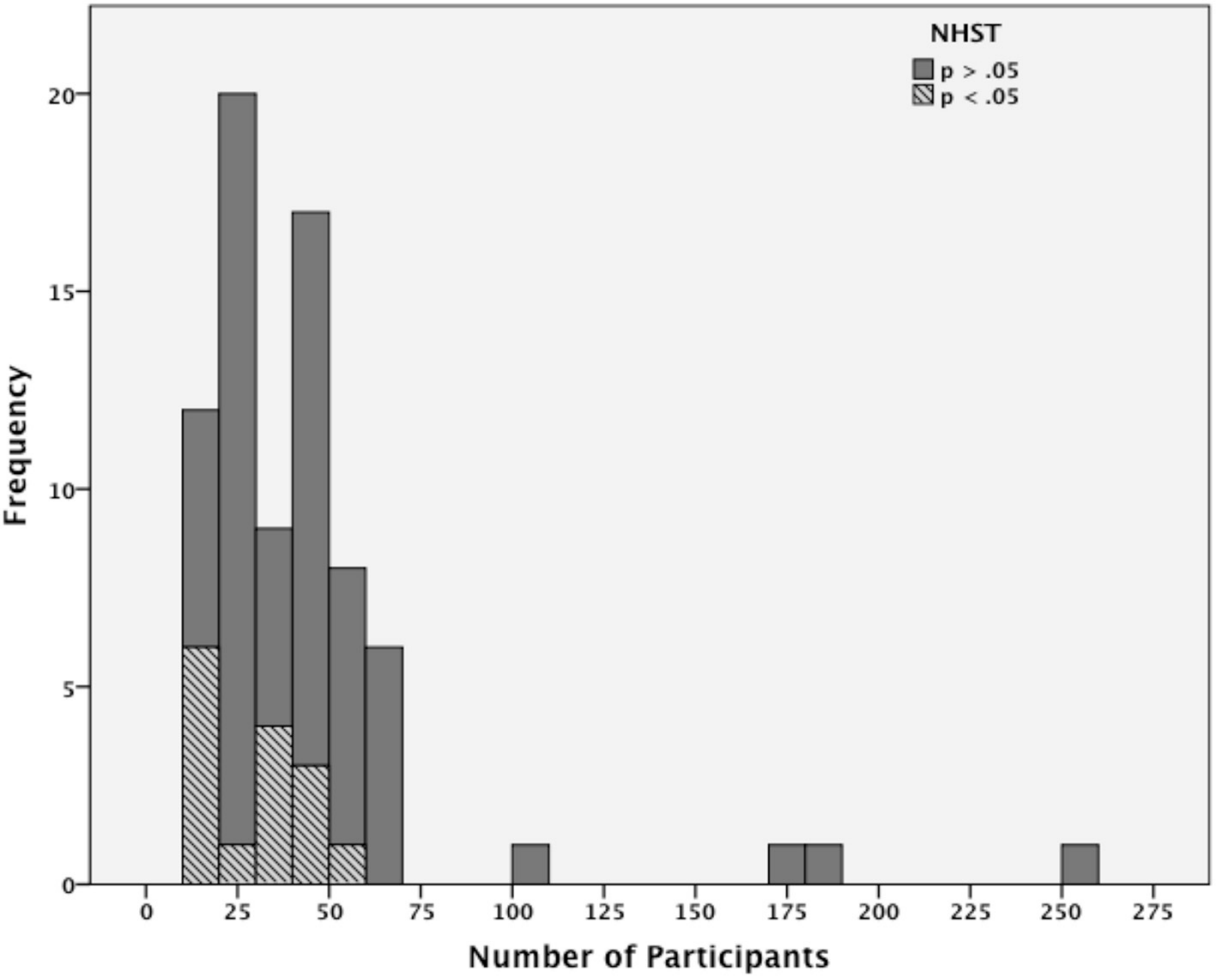
**Frequency of significant (*p* < 05) and non-significant (*p* > 0.05) bilingual advantages for different numbers of participants per language group**. The histogram is based on Paap et al. (2014) appendix that collated tests of either inhibitory control or monitoring in non-verbal interference or switching tasks frequently used to measure executive functioning. The tests are drawn from 35 reports appearing outside Hilchey and Klein's (2011) review and includes 76 individual tests.

Paap et al. (2014) tabulated 76 tests for bilingual advantages appearing outside Hilchey and Klein's (2011) review that includes the 30 studies analyzed by Hilchey et al. (2014). The tests listed by Paap et al. (2014) come from 35 different reports that used either non-verbal interference tasks or non-verbal switching tasks and derived measures typically associated with either inhibitory control or conflict monitoring in non-verbal interference tasks and either switching or mixing costs in non-verbal switching tasks. Figure 2 is a histogram constructed from the studies listed by Paap et al. (2014) showing the total of significant and non-significant results as the number of participants per language group increases. It is clear by visual inspection that since the Hilchey and Klein review in 2011, that bilingual advantages tend to occur when there are a small number of participants per language group whereas null results occur both with small n and large n.

Small n's reduce an experimental design's power to correctly reject the null hypothesis, but as Bakker et al. (2012) demonstrate with simulations, small n's coupled with a bias against null findings also results in an inflated rate of false positives. The *European Journal of Personality* in its recent recommendations for increasing replicability in psychological science urges increases in sample size and the avoidance of multiple underpowered studies (Asendorpf et al., 2013). If the effect of bilingualism on EF was generously estimated to be of medium size (Cohen's *d* = 0.5), if the effect was tested with an alpha of 0.05, and if a researcher was willing to accept a power of only 0.67, then one would need 36 participants in each of two language groups given a one-tailed test and 48 in each group for a two-tailed test. Francis (2012) bluntly asserted that “Studies with unnecessarily small sample sizes should not be published” (p. 989). The specific role of small n's coupled with confirmation bias has been discussed in Paap and Liu (2014) and Paap (2014).

### Large samples sizes and “ideal” bilinguals

Given the reasonable (although debatable) conjecture that benefits of bilingualism are likely to develop to a maximum in bilinguals who are highly proficient, acquire both languages early, and reside in language communities where most people speak the same two languages and switching is ubiquitous; the studies by Duñabeitia et al. (2014), Antón et al. (2014), and Gathercole et al. (2014) deserve special attention. Duñabeitia et al. (2014) compared Spanish monolinguals (*n* = 252) to Basque-Spanish bilinguals (*n* = 252) at six successive grades with respect to both a verbal Stroop task and a number-size congruency task. Bilinguals and monolinguals performed equivalently in these two tasks in terms of global RT and across all the indices of inhibitory control explored across all grade levels. Antón et al. compared a group of 180 Basque-Spanish bilingual children with a group of 180 carefully matched monolinguals on an ANT version of the flanker task. The comparison between the language groups was consistent and null: no inhibitory advantage (incongruent-congruent), no global RT advantage, no alerting advantage, and no orienting advantage. The Gathercole et al. study of Welsh-English bilinguals was a lifespan study testing seven age groups (from 3 years of age through over 60). They reported no systematic language-group differences on three tasks assumed to reflect EF: dimensional card sorting (*N* = 650), Simon (*N* = 557), and a grammaticality judgment with irrelevant semantic anomalies (*N* = 354). All three studies share the strengths of using bilinguals immersed in a bilingual region, monolingual control groups from the same country, a very large number of participants, multiple age groups, and multiple measures of EF. In summary, the many recent failures to find language-group differences strains to the breaking point the conclusion that managing two languages *consistently* leads to performance advantages favoring bilinguals. On the other hand, these failures do not preclude the possibility that the cumulative research enterprise will eventually hone in and identify the specific aspects of managing two languages that enhance specific components of EF.

## Convergent validity

### Measures of interference control

The replicability problem is compounded by the fact that measures and tasks typically used to demonstrate bilingual advantages appear to lack convergent validity. This may be viewed as surprising given the preceding discussion of Miyake and Friedman's work, but there are subtle differences between their work and research reporting bilingual advantages. Most tests for a bilingual advantage in EF in adults have focused on only two of the three components studied by Miyake and Friedman (viz., switching and inhibition, but not updating). Furthermore the early reports of bilingual advantages often used the Simon task, a task that was never included in any of the Miyake and Friedman studies using confirmatory factor analysis. It should also be noted that Miyake and Friedman report that the latent variable for inhibition is not separable and that the factor loading from specific interference tasks (i.e., the observed measures) to the inhibition factor are weaker than those observed for updating and switching.

When two or more measures of inhibitory control are tested in the same experiment the cross-task correlations are often not significant. Paap and Greenberg (2013) discuss five studies (Fan et al., 2003; Stins et al., 2005; Humphrey and Valian, 2012; Kousaie and Phillips, 2012) that yielded 10 non-significant cross-task correlations and a structural equation study that found no significant association between the flanker and Simon task (Keye et al., 2009). In their own work Paap and Greenberg (2013) reported near zero correlations between RTs on antisaccade trials and the magnitude of the Simon effect (Study 1, *r* = −0.12) and between the magnitude of the Simon and flanker effects (Study 3, *r* = −0.01). Shilling et al. (2002) reported that all six pairwise correlations between four variants of the Stroop task ^4^ were non-significant with r's ranging from −0.13 to +0.22, *n* = 49. Similarly, the correlation between the standard Stroop and the non-verbal Stroop (number-size congruency) used by Duñabeitia et al. was small, *r* = +0.14; albeit significant, *p* < 0.05, with an *n* of 504.

A somewhat more promising picture arises from studies by Unsworth and colleagues (Unsworth et al., 2009, 2012, 2014; Unsworth and Spillers, 2010) who used latent variable techniques to assess the relationship between attentional control (AC), working memory capacity (WM), secondary memory (SM), and general fluid intelligence (gF). These relationships are represented in blue in Figure 1. The AC construct was tested using four tasks that they view as requiring either constraining (arrow flanker), restraining (antisaccade and Stroop), or sustaining (psychomotor vigilance) attention. The first two categories of AC (constraining vs. restraining) honor the traditional distinction between interference control (suppression of interference due to stimulus competition) and response inhibition (suppression of prepotent responses)^5^. In other words, the AC construct in the Unsworth studies approximates the inhibition construct in the Miyake and Friedman studies. Thus, when comparing and contrasting these studies we will often refer to the “AC-Inhibition” factor.

The correlations between the tasks forced to load on the AC-Inhibition factor in the Unsworth studies were relatively small, but always significant. More specifically across three studies, the correlations between antisaccade accuracy and flanker interference were all significant (*p*'s < 0.05) and ranged from −0.25 to −0.35. Only one of the studies (Unsworth and Spillers, 2010) measured Stroop interference and that measure significantly correlated with both antisaccade accuracy, *r* = −0.15, *p* < 0.05, and flanker interference, *r* = 0.17, *p* < 0.05, with *n*'s of 181.

These small, but significant cross-task correlations mirror the Miyake and Friedman findings, but contrast with the many studies reviewed in the preceding section that showed non-significant cross-task correlations. Perhaps a fair summary of the review to this point is that individual tasks assumed to measure inhibitory control tend to show weak (at best) convergent validity with one another, while at the same time the latent variable for the inhibiting component is consistently related to the updating and switching components or to EF as a unitary construct. It is somewhat unsettling that interference control appears to be, on the one hand, the glue that holds the EF construct together while, on the other hand, resisting all attempts to find a gold standard or benchmark task that can be used as a general measure of inhibitory control.

### Measures of updating and WM

The confirmatory factor analyses reported by both Miyake and Friedman and Unsworth's group (2010) include a construct intended to represent the controlled manipulation of information in primary memory. Miyake and Friedman refer to this construct as Updating and typically use these tasks: letter-memory, keep-track of the last instance of several semantic categories, and visual memory for the spatial location of objects appearing two trials back. Across the Miyake and Friedman studies there is strong evidence for convergent validity: the mean factor loading for a total of nine Updating tasks was +0.56. Zero-order correlations are not always reported, but range from +0.28 to + 0.41.

In contrast, the Unsworth group refers to their construct as WM and use the classic “storage and processing” tasks developed by Engle (2001): reading span, operations span, counting span, and symmetry span^6^. The span tasks also show good convergent validity with each other. Although the symmetry span task consistently showed the lowest factor loading, the mean factor loading of 13 span measures across five different studies was +0.75. Similarly, the mean zero-order correlations between 11 pairs of span tasks was +0.57. There appears to be very good convergent validity among the WM span tasks, perhaps even better than the impressive factor loadings and cross-task correlations reported for the Updating tasks.

Although one might expect that Updating and WM are the same construct and provide similar measures of the ability to manipulate information in primary memory, the existing evidence suggests only a moderate relationship. Engle et al. (1999) reported that the correlations between the keep track (an Updating measure) and three WM span tasks ranged from +0.22 to + 0.36. Similarly, Miyake et al. (2000) reported that the correlations between the Ospan (a WM measure) and three Updating tasks ranged from +0.28 to +0.41. To use Miyake and Friedman's terms, WM and Updating are related, but appear to be quite separable.

The treatment of WM in the literature on bilingual advantages in EF has varied in important ways. To take just one example, Prior and MacWhinney (2010) in their seminal test for differences in switch costs use the operations span task as a matching variable to demonstrate that their samples of monolinguals and bilinguals were not confounded by differences in WM. Although Prior and MacWhinney do not elaborate on their treatment of WM, casting WM in the role of a control variable implies that it is a potential “mediator” (Baron and Kenny, 1986) that could provide an alternative causal explanation for the association between bilingualism and EF ability. This seems too simplistic because the confirmatory factor analyses reported by the Unsworth group in all four studies showed that the latent variable for WM is highly related to the AC-Inhibition latent variable (mean *r* = 0.54). Thus, if instead of viewing WM as a mediator, it is treated as one of the core components of shared EF, then one would expect that advantages in inhibitory control to often be accompanied by advantages in WM. A similar logic led Ratiu and Azuma (2012) to compare 52 Spanish-English bilinguals to 53 English monolinguals on four different WM tasks. Ratiu and Azuma reported no bilingual advantages in any of the four tasks, including the non-verbal symmetry-span task.

### Measures of monitoring

The current trend in the literature on bilingual advantages is to appeal to monitoring (e.g., Costa et al., 2008) or loosely defined constructs such as *coordination* or *mental flexibility* (e.g., Kroll and Bialystok, 2013) as the essence of the bilingual advantage in EF. Monitoring is often described as the ability to monitor for goal-relevant information and/or detect conflict from competing information that may become the target for inhibition. Global RT (the average across both congruent and incongruent trials) or simply the mean RT on congruent trials is often used as a measure of monitoring. A better test for a “monitoring” advantage would compare the mean RT on congruent trials from a mixed block to a baseline RT consisting of trials where conflict never occurs. Yet another common measure of monitoring ability is the mixing-cost measure computed from switching tasks. Across a variety of measures the overall pattern of correlations reviewed by Paap and Greenberg (2013) showed no convergent validity.

## Purpose

Multiple tasks and measures of EF are rarely included in the same test for bilingual advantages in EF. The present study enables the derivation of 13 different measures of EF from four common non-verbal tasks: antisaccade, color-shape switching, Simon, and ANT. Language group differences are more compelling if they significantly appear in more than a single task. A second important goal was to assess the convergent validity through cross-task correlations of indices assumed to measure the same component of EF.

## Method

### Participants

The 120 participants were San Francisco State University (SFSU) students who participated in order to fulfill a class requirement or for extra credit. The study was approved by the SFSU IRB. The vast majority were junior and senior psychology majors. Proficiency in a spoken language was self-rated using the 7-point scale described in Paap and Greenberg (2013) and we used the same criteria to classify participants as bilinguals (viz. a proficiency of 4 or more in at least two languages) or monolinguals.

#### Language characteristics

Table 1 shows the basic language characteristics of the two language groups participating in the present study. The mean proficiency in English for both groups was well over 6 and the median and mode for both groups was 7. For the bilinguals the mean proficiency in their other language was 5.7 with a median and mode of 6.0. A rating of 6 represents *Fluent: as good as a typical native speaker* and a rating of 7 represents *Super Fluency: better than a typical native speaker*. As a group our bilinguals are highly fluent in at least two languages and 25% are fluent in three or more languages.

**Table 1:** **Language characteristics of monolinguals and bilinguals: mean (SE)**.

**Group**	***N***	**English Pro**.	**Other Pro**.	**English reading**	**English AoA**	**Other L AoA**	**% English use**	**Switch frequency**
Bilinguals	58	6.3 (0.12)	5.7 (0.18)	3.8 (0.13)	3.9 (0.64)	2.3 (0.75)	70.1 (2.9)	2.8 (0.19)
Monolinguals	62	6.6 (0.07)	1.3 (0.18)	3.9 (0.10)	0.2 (0.12)	9.0 (1.0)	96.8 (1.2)	0.4 (0.10)

*n, sample size; SD, standard deviation; Pro., proficiency; AoA, age of acquisition*.

Of the total set of 58 bilinguals 16 are native speakers of both English and one other language, 10 are native speakers of English and acquired another language as an L2, and the remaining 32 acquired English as an L2 and are native speakers of a language other than English. The median age-of-acquisition for the bilinguals who had only one native language and acquired an L2 was 6.0 years of age. In addition to English our bilingual group included fluent speakers of Spanish (35), Vietnamese (6), French (6), Cantonese (5), Hindi (5), Urdu (4), Punjabi (3), Tagalog (2), Russian (2), Mandarin (2), Arabic (1), Bulgarian (1), Farsi (1), German (1), Greek (1), and Italian (1). Slightly over half of our bilinguals acquired English as an L2, but even this subset of bilinguals rate their English proficiency as 5.9 on average.

Our bilinguals are full-time students at a university where English is the language of instruction and consequently spend substantial amounts of time producing and comprehending English. Despite their role as students, almost all of our bilinguals currently use both languages every day. They report speaking English 70% of the time.

When asked to report their frequency-of-switching on a 5-point scale the median and modal response was 3: *a couple of times a day*. We also asked the bilinguals to estimate the percentage of time spent thinking in English vs. the other languages they knew. Only one bilingual, whose native language was Vietnamese, reported thinking exclusively in English.

Some researchers are skeptical about the accuracy of self-ratings of language proficiency, but self-ratings are highly correlated with a range of objective and standardized measures of language proficiency. For example, a study by Marian et al. (2007) correlated self-report measures of reading, speaking, and listening proficiency with eight different standardized measures of language skill involving reading, writing, speaking, and listening and covering both comprehension and production. These correlations were obtained for both L1 and L2 where L1 was defined as the language a bilingual acquired first. For L2 (the proficiency of greatest concern in classifying an individual as bilingual), all 24 correlations between the three subjective measures and the eight objective measures were significant with Pearson r values ranging from 0.29 to 0.74 with a mean of 0.59. Taking all of their results into account Marian et al. concluded that self-ratings are *“an effective, efficient, valid, and reliable tool for assessing bilingual language status.”* (p. 960). In a similar study Francis and Strobach (2013) reported that self-ratings in both English and Spanish are highly predictive of standardized objective measures.

In other studies conducted in our lab (Paap and Greenberg, 2013; Paap and Liu, 2014) using the same population of student participants and the same recruiting methods self-rated English proficiency significantly predicted performance in: (a) a sentence comprehension task requiring resolution of lexical ambiguity (b) judging if sentences contain a semantic anomaly, (c) judging if sentences contain a syntactic error, (d) judging if letter strings are English words or non-words, (e) category fluency (number of correct responses to a category probe), and (f) reading time to critical word in sentences with a semantic anomaly or syntactic error.

#### Demographic characteristics

Table 2 shows the means and standard deviations for the two language groups on six characteristics that are not related to language, but that may influence task performance. These include the level of education of the participant's most highly educated parent (PED) and age. The measure Frequency Multitasking is a composite of responses to four items from our background questionnaire that tap into the individual's multitasking experiences. Another characteristic shown in Table 2 is a self-rating on a 5-point scale of the degree to which the individual excels at team sports. The final characteristic assesses the individual's attitude toward multitasking rather than the frequency of actual behaviors. The differences between the means for bilinguals and monolinguals on each of these six characteristics were evaluated with a set of *t*-tests. Five of the mean differences were negligible and yielded *p*'s > 0.55. There were marginal differences for PED.

**Table 2:** **Other characteristics of bilinguals and monolinguals in Experiment 2: mean (SE)**.

**Group**	**PED**	**Age**	**Frequency multitasking**	**Excel team sports**	**Attitude multitasking**
Bilingual	3.7 (0.23)	24.4 (0.78)	14.8 (0.55)	2.4 (0.15)	2.4 (0.15)
Monolingual	4.3 (0.19)	24.8 (1.1)	14.4 (0.50)	2.5 (0.14)	2.4 (0.13)

*PED, parent's educational level*.

PED information was obtained with a six-point rating scale where level 3 represents *attended college, but did not graduate* and level 4 represents *earned an associate of arts or other two-year degree*. The mean PED score for the bilinguals (3.71) was smaller than that for the monolinguals (4.29), but the difference was not significant using the standard alpha level of 0.05, *t*_(111)_ = −1.96, *p* = 0.053. This potential problem will be thoroughly addressed later, but the short version is that across a very large sample of SFSU students the correlation between PED and several measures of EF ability are non-significant and usually near zero.

### Simon task

The Simon task was identical to the one used in Studies 2 and 3 by Paap and Greenberg (2013).

#### Trial definition

Each trial began with the presentation of a center fixation (+) for 500 ms. The center fixation was immediately followed by the target stimulus which was either a “Z” or a “/.” The participant's task was to press the corresponding key as quickly as possible without making errors. The left index finger rested on the “Z” key and the right index finger rested on the “/” key. In a neutral block the target was displayed either 2.3° above or below the center fixation. In a Simon block the target was displayed either 3.9° to the left or to the right of the center fixation. In a Simon block a trial was defined as congruent if the location of the target was on the same side as the correct response and as incongruent if the location of the target was on the opposite side.

#### Design

The critical Simon blocks were always the last two of four blocks. Each Simon block consisted of 20 congruent trials and 20 incongruent trials presented in random order. Half the trials of each type presented the target on the left with the other half presented the target on the right. Thus, the mean response time (RT) for the four conditions defined by the combination of two blocks and two levels of congruency (congruent vs. incongruent) were each based on 20 trials and when collapsed across blocks of 40 trials. In the first two blocks of trials the target was displaced either above or below the center fixation. This creates a “neutral” condition because the location of the target is neither compatible nor incompatible with pressing the “Z” key on the left or the “/” key on the right. Block 1 provided 20 trials of practice in the neutral condition and was followed by a 40-trial Block 2.

### Color-shape switching task

The color-shape switching task was identical to that used by Paap and Greenberg (2013) in Studies 1–3. The task was patterned on that used by Prior and MacWhinney (2010).

#### Trial definition

Each trial began with the presentation of a center fixation (+) for 350 ms and then a blank screen for 150 ms. The left middle and index fingers rested on the “Z” and “X” key, respectively. The right index and middle fingers rested on the “.” and “/” keys, respectively. In a pure color block the participant's task was to press the “Z” key if the target was blue and the “X” key if it was red. In a pure shape block the task was to press the “.” key if the target was a circle and the “/” key if it was a triangle. The target set consisted of a blue circle, a blue triangle, a red circle, and a red triangle.

In a mixed block the target was preceded by a precue for 250 ms that remained in view until the participant responded to the target. If the precue was a rainbow then the participant had to make a color decision when the target appeared. If the precue was a black circle embedded within a black triangle then the participant had to make a shape decision when the target appeared. Participants were instructed to respond as quickly as they could on the basis of the precued dimension (viz., color or shape). Each trial was designated as a “repeat” trial if the cued decision was the same as on the previous trial and a “switch” trial if it was different. Each target and precue subtended about 1.83° of visual angle with the center of the precue appearing 2.3° above the center of the fixation stimulus and the upcoming target.

#### Design

The task consisted of six blocks. The first block of 16 trials was “pure” color. Each of the four targets appeared four times in random order. The second block of 16 trials was “pure” shape with each of the targets appeared in random order. Following Block 2 the “mixed” task was introduced with detailed instructions regarding the use of the precue to signal whether a color or shape would be required on each specific trial. Each of the four “mixed” blocks started with two buffer trials that were not analyzed. Block 3 was a practice block and consisted of 18 trials (including the two buffers). Blocks 4–6 each consisted of 50 trials (including the two buffers). A single random order was used for every participant. Each of the four targets appeared 36 times across Blocks 4–6 and there were 72 repeat trials and 72 switch trials.

### Antisaccade task

The task was identical to that used by Paap and Greenberg in their Study 1. The design, materials, and procedure for the antisaccade task were closely modeled from those used by Kane et al. (2001).

#### Trial definition

Experimental trials consisted of the following sequence of events: (1) a center fixation (^***^) was presented for a variable duration (i.e., 600, 1000, 1400, 1820, 2200 ms) in order to introduce temporal uncertainty; (2) a blank field for 100 ms; (3) a “#” sign for 100 ms displaced 2° to the opposite side from the eventual target; (4) a blank field for 50 ms; (5) the “#” sign in the same location for 100 ms; (6) a target letter (“B,” “P,” or “R”) for 150 ms displaced a comparable extent on the opposite side; (7) a mask (“8”) presented until the response. The target and mask subtended about 0.9° of visual angle. The task on each trial was to identify the target stimulus (i.e., “B,” “P,” or “R”) by pressing the key with the corresponding label using three fingers of the right hand.

The baseline trials presented no opposite field distracter and consisted of these events: (1) a center fixation (^***^) was presented for a variable duration (i.e., 600, 1000, 1400, 1820, 220 ms); (2) a blank field for 100 ms; (3) a centered target-letter (“B,” “P,” or “R”) for 150 ms; and (4) a mask (“8”) presented until the response.

#### Design

The antisaccade trials were preceded by a block of control trials that used a centered target and no distracting stimulus. The control trials provided a baseline response time (RT) that should require little or no EF. The trials were organized and presented in the following order. A practice block consisted of 15 baseline trials, one at each combination of 5 fixation durations and 3 target letters and presented in random order. Block 2 was identical to the first block and provided the baseline RTs. Block 3 was 30 anti-saccade trials formed by the random combination of: 5 fixation durations by 3 target letters by 2 sides (left and right).

### ANT task

The ANT task was similar to that developed by Fan et al. (2002) and identical to the one used by Paap and Greenberg (2013).

#### Trial definition

The congruent display consisted of a central arrow pointing either left or right and two flankers on each side pointing in the same direction. A single arrow subtended about 0.9° of visual angle and the entire horizontal extent of the five-arrow stimulus was about 6.3°. In the incongruent displays the flankers pointed in the opposite direction from the central target arrow. The sequence of events was as follows: (a) a fixation point (a plus sign) appeared at the center of the screen and remained throughout the trial, (b) a cue (described below) was presented for 100 ms, (c) followed by the fixation field for an additional 400 ms, and then (d) the target display until the participant's response or for up to 1700 ms. The target was vertically displaced either 1.2° above or below the fixation point. Participants were instructed to press the “z” key with their left index finger if the target arrow pointed left and to press the “/” key with their right index finger if the target arrow pointed right.

Consistent with the ANT methodology four types of cues were used. On “no cue” trials the 100 ms cue display is simply a continuation of the centered fixation point (+). Obviously it affords no information about the temporal onset or spatial location of the upcoming target. The “double cue” display consists of a two ⋄ symbols above and below the fixation point. This provides no information about the location of the upcoming target, but does reduce the temporal uncertainty. Subtracting the means of the double cue trials from the no cue trials yields the alerting effect. The third type of cue is the “central cue” that simply replaces the + fixation point with the ⋄ symbol. It does reduce temporal uncertainty, but provides no cue to spatial location. In contrast, the “spatial cue” display adds a valid diamond cue above or below the fixation point. As both the “central cue” and “spatial cue” displays provide the same advantages in alerting, the mean of the “spatial cue” trials can be subtracted from the mean of “central cue” trials to derive the orienting effect.

#### Design

Block 1 consisted of 20 neutral trials where all the targets consisted of a centered arrow and the flankers were dashes. Each target was randomly preceded by one of the four cue types. Block 1 is similar to the block of neutral trials that initiated the Simon task and, likewise, enables the computation of mixing costs by subtracting the mean of these neutral trials from the mean of the congruent trials in the experimental blocks that randomly mix conflict and no-conflict trials.

Blocks 2 through 5 were standard ANT blocks with 50% congruent and incongruent trials. Block 2 consisted of 16 trials and was considered practice. Blocks 3–5 each consisted of 64 trials with 8 repetitions of the combinations formed by 2 target types (congruent vs. incongruent) × 4 cue displays. Thus, given standard practice for analyzing each attentional network (executive attention, alerting, and orientating) in the ANT each block provided 32 trials of each condition (e.g., 32 congruent and 32 incongruent trials) and overall means were based on 96 trials. The trials within each block were randomized.

## Results

### Definition of 13 measures of EF

Table 3 shows the 13 measures of EF that were computed for each participant from performance across the four tasks. For each measure both the common name (e.g., flanker effect) and the operational definition (e.g., mean RT incongruent trials − mean RT congruent trials) are provided. Also shown is the block to block reliability for each measure.

**Table 3:** **Block to block reliability of 13 assumed measures of EF**.

**Task**	**Operational Definition**	**Trials per condition**		**SBP**	***p***
**Measure**		**1st**	**2nd**		
**ANTISACCADE**					
RT	Mean RT of all antisaccade trials	60		0.90	<0.01
RT cost	Mean RT anti − mean baseline	60	30	0.53	<0.01
PC	Mean PC of all antisaccade trials	60		0.94	<0.01
PC cost	Mean PC − mean baseline	60	30	0.72	<0.01
**FLANKER**					
Effect	Mean RT incongruent − congruent	96	96	0.75	<0.01
Mixing costs	Mean RT congruent − mean baseline	96	20	0.92	<0.01
Global RT	Mean RT across congruent and incongruent	192		0.89	<0.01
Shifting cost	Mean RT shift trials − repeat trials	96		0.82	<0.01
**SIMON**					
Effect	Mean RT incongruent − congruent	40	40	0.65	<0.01
Mixing costs	Mean RT congruent − mean baseline	40	20	0.70	<0.01
Global RT	Mean RT across congruent and incongruent	80		0.93	<0.01
**SWITCHING**					
Switch cost	Mean RT switch trials − repeat trials	48	48	0.78	<0.01
Mixing cost	Mean RT repeat trials − pure trials	48	32	0.79	<0.01

*PC, Proportion Correct; SBP, Spearman-Brown Prophecy correlation; p, exact probability. SBP for Flanker measures are averages of Block 1 to Block 2, Block 2 to Block 3, and Block 1 to Block 3*.

#### Antisaccade task

The mean RTs in a pure block of antisaccade trials has been used as a measure of inhibitory control. Because our design included a block of baseline trials where there was no distractor and the targets were presented at fixation, a second measure of inhibitory control subtracts the mean RT on the baseline trials from the mean RT on antisaccade trials. The measure is referred to as *antisaccade costs*. Because the primary dependent variable in antisaccade trials is often accuracy (e.g., the Unsworth studies), two additional measures were derived from the antisaccade task using proportion correct rather than RT.

#### Simon and ANT task

Three similar measures of RT were derived for both the Simon and ANT task. For each task a measure of inhibitory control was defined as the difference in mean RT between the congruent and incongruent trials (i.e., the standard Simon/flanker interference effect). Despite the acute impurity problem, global RT (the mean RT across both the congruent and incongruent trials) has often been used as measure of monitoring and for continuity we also treat global RT as a measure of EF. An arguably more pure measure of monitoring subtracts the mean RT of a baseline condition from the mean of the congruent trials (from a block that randomly mixes congruent and incongruent trials). For the Simon task the baseline condition is a block of trials where the targets are displaced above or below fixation rather than to the left and right. For the flanker effect the baseline conditions is a block of trials where the flankers are dashes rather than arrows. These two measures of monitoring are referred to as flanker *mixing costs* and Simon *mixing costs*.

Costa et al. (2008) also reported bilingual advantages in *shifting costs* in their ANT task and this measure was also computed in our study. Shifting costs are the context or sequential dependency effects that occur in the mixed block where congruent (C) and incongruent (I) trials are randomly presented. The congruency of the current trial is either the same as the previous trial (represented as cC or iI) or different (cI or iC). The *shifting cost* measure is the differences between trials that require shifting (cI and iC) compared to no-shift trials (cC and iI).

#### Color-shape switching task

Two measures of EF are derived from the color-shape switching task. The differences between the repeat trials and switch trials from the block where the required decision is precued during each trial are referred to as *switching costs* and are usually assumed to reflect the efficacy of the ability to switch^7^. The second measure is the difference between the mean of the single task (pure color or pure shape) trials and the repeat trials from the mixed block. This difference is referred to as *mixing costs* and is usually assumed to provide a measure of the monitoring component (including preparation for a possible switch) of EF.

## Results on the effects of bilingualism

The RT analyses were based only on trials with correct responses. The standard deviation (SD) for the experimental trials of each individual participant were computed and RTs that exceeded 2.5 SDs were trimmed (Ratcliff, 1993). In all cases there were less than 2.7% trimmed responses. Three participants (2 bilingual, 1 monolingual) were deleted from the analyses of the antisaccade task because their accuracy levels were near chance levels. No participants were removed for performance reasons in any of the other three tasks.

### Main effects for difference score measures

Nine of the 13 measures shown in Table 3 are differences scores. For each a dependent measures *t*-test (collapsed across language groups) compared the mean for the “difficult” condition to that for the “easy.” For example, the overall flanker effect was 85 ms based on a mean of 593 ms on the incongruent trials compared to only 508 on the congruent trials. The condition means, standard errors, t values, and the exact probabilities are shown in Table 4. Seven of the 9 differences are highly significant with *p* < 0.001. The mean proportion correct on the antisaccade trials was not significantly different from that on the block of neutral trials and overall accuracy was at 90%. Based on Paap and Greenberg the primary dependent measure for this instantiation of antisaccade costs should be RT rather than accuracy and the RT costs were highly significant.

**Table 4:** ***T*-tests for main effects of trial type**.

**Task**	**Easy**		**Difficult**		**Diff**.	***t***	***p***
**Measure**	**Mean**	***SE***	**Mean**	***SE***			
**ANTISACCADE**							
RT cost	564	19.62	610	19.86	46	4.19	<0.001
PC cost	0.900	0.017	0.909	0.014	−0.013	−1.51	0.134
**FLANKER**							
Effect	508	6.64	593	7.28	85	32.58	<0.001
Mixing costs	484	7.18	508	6.64	24	4.40	<0.001
Shifting cost	542	7.04	553	7.00	10	5.64	<0.001
**SIMON**							
Effect	467	8.92	499	7.48	32	8.46	<0.001
Mixing costs	470	8.00	468	9.38	−2	0.61	0.545
**SWITCHING**							
Switch cost	819	30.94	1026	37.61	206	15.75	<0.001
Mixing cost	567	19.07	819	30.94	253	10.05	<0.001

*PC, Proportion Correct; SE, standard error; Diff., difficult mean—easy mean; t, obtained t statistic; p, exact probability*.

The only problematical outcome is the Simon mixing costs in that the RTs on the neutral block and those on the congruent trials from the mixed block are nearly identical. If conflict monitoring is required in the mixed block, but not in the neutral block, then there should be longer RTs for the congruent trials in the mixed block. The neutral block displaced the target above and below the fixation rather than to the left or right. Although the vertical displacement eliminates the conflict between, for example, a spatial location on the left and a more distal and incompatible correct response by the right hand; shifting attention up or down may be more difficult than shifting attention to the left or right. The mixed block may also have benefited from additional practice. In any event, the fact that mixing costs in the Simon task had a mean near zero does not imply that it could not serve as a good measure of individual differences in monitoring. That is, participants with positive differences may be better monitors than those with negative differences. This result for mixing costs in the Simon task is not due to an unusually weak instantiation of the basic Simon task as the 32 ms main effect is in the precise interval that Lu and Proctor (1995) characterize as the typical Simon effect ^8^.

### Simple *t*-tests for the 13 measures of EF

Table 5 shows the results of independent group *t*-tests for each of the 13 measures of EF. Six of the 13 are in the direction of a bilingual advantage, but the t statistics for this subset are all non-significant with p values ≥ 0.09. Three of the measures showed a significant monolingual advantage: antisaccade RT (*p* = 0.027), Simon global RT (*p* = 0.006), and the Simon effect (*p* = 0.006). A monolingual advantage for mixing costs in the switching task might be considered marginally significant (*p* = 0.105), as might the bilingual advantage in the flanker effect (*p* = 0.090).

**Table 5:** ***T*-tests for language group differences for the 13 measures of EF**.

**Task**	**Bilingual**		**Monolingual**		**Diff**.	***t***	***p***
**Measure**	**Mean**	***SE***	**Mean**	***SE***			
**ANTISACCADE**							
RT	657	36	567	19	+90	2.25	0.027
RT cost	34	17	41	11	−7	−0.36	0.720
PC	0.916	0.019	0.928	0.015	−0.013	−0.52	0.603
PC cost	0.004	0.009	0.022	0.015	−0.018	−1.01	0.317
**FLANKER**							
Effect	81	3.84	89	3.46	−8.9	−1.71	0.090
Mixing costs	21	7.19	26	8.10	−4.8	−0.45	0.655
Global RT	558	9.59	537	9.76	20.5	1.50	0.136
Shifting cost	12	2.44	9	2.81	3.1	0.84	0.404
**SIMON**							
Effect	41	3.79	28	3.50	13.2	2.57	0.012
Mixing costs	−5	5.00	−4	5.16	−0.6	−0.09	0.931
Global RT	490	8.21	463	5.41	27.4	2.80	0.006
**SWITCHING**							
Switch cost	200	18.21	212	18.90	−12.4	−0.47	0.638
Mixing cost	294	41.51	212	28.09	81.7	1.64	0.105

*PC, Proportion correct; SE, standard error; Diff., bilingual mean—monolingual mean; t, obtained t statistic; p, exact probability*.

### Regression analyses using L2/L1 balance and PED as predictors

As reported earlier and shown in Table 2 our monolinguals have significantly higher PED scores compared to the bilinguals. However, PED does not predict performance on any of the 13 measures of EF. The largest correlation is between PED and RT on antisaccade trials, *r* = −0.12, *p* = 0.232. All others are smaller than *r* = ±0.10 and six are within ±0.05 of zero. The absence of PED associations was also reported by Paap and Greenberg (2013) drawing samples from the same student population. For example, combined samples of 267 participants yielded *r*'s of +0.042, +0.014, and −0.005 for PED and the Simon effect, switching costs, and mixing costs, respectively. Paap and Greenberg also formed subsets of monolinguals and bilinguals that were precisely matched on PED scores and reported no differences compared to the full set for any of the measures they tested. Thus, there is no evidence to support the possibility that the absence of bilingual advantages in the present study is due to group differences in PED. As a final check, PED is included as a predictor in the regression analyses reported next.

Kroll and Bialystok (2013) observe that it may be statistically advantageous and conceptually superior to use a continuous measure of bilingualism rather than a dichotomy. Consequently in the following regression analyses we used a balance measure of bilingualism that is very similar to the one used by Bialystok and Barac (2012). Balance was computed for each participant as the ratio of minimum proficiency to maximum proficiency. For example, a bilingual with a proficiency of 5 in English and 7 in Cantonese would have a balance score of 5/7 or 0.71. A monolingual with a proficiency of 1 in French and 7 in English would have a balance score of 0.14. Given this operational definition the range of balance scores is from 0 to 1.

In order to statistically control for differences in PED each of the 13 measures was used as an outcome variable in a regression analysis that included both balance and PED as predictors. The standardized beta coefficient for the balance predictor was significant in only 1 of the 13 models, the one predicting the magnitude of the Simon effect, β = +0.217, *t* = 2.244, *p* = 0.027. The positive β coefficients indicates that as the balance score increases, the magnitude of the Simon effect increases. This, of course, reflects a bilingual disadvantage, not an advantage. The two monolingual advantages revealed by *t*-tests comparing group means (viz. antisaccade latency and Simon global RT) and the marginally significant bilingual advantage with the flanker-effect measure vanished in the regression analysis. On balance there is no coherent evidence for language-group differences across the 13 measures with the possible exception of the monolingual advantage with the Simon effect.

### Regression analyses using five demographic predictors

For each of the 13 measures a stepwise regression analysis was performed using the following predictors: chronological age, video gaming frequency, frequency of multitasking, attitude toward multitasking environments, and ability at team sports. Eleven of 13 analyses yielded empty models and the other two consisted of a single significant predictor: frequency of video gaming for antisaccade RT costs (β = −0.248, *t* = −2.20, *p* = 0.030) and attitude toward multitasking for Simon global RT (β = −0.208, *t* = −2.029, *p* = 0.045). The negative coefficients are consistent with the expectation that EF skills would be higher for gamers and those with positive attitudes toward multitasking, but perhaps the main message is that these demographic variables are poor predictors of individual differences in EF.

## Discussion of effects of bilingualism

All of the tasks and measures used in the present study were identical to those used by Paap and Greenberg (2013) and the participants were drawn from the same participant pool. With respect to bilingual advantages the present and previous studies are completely consistent: there were no statistically-significant (*p* < 0.05) bilingual advantages. A puzzling finding was that the monolingual advantage for the Simon effect reported by Paap and Greenberg (2013) in their Study 3 and in their analysis of the combined data from Studies 1 to 3 was replicated in the present study in both the independent-groups *t*-test and in the regression analysis that includes PED as an additional predictor. The combined data from our lab suggests that there is a small monolingual advantage in the magnitude of the Simon interference effect. The overall Simon effect observed in Paap and Greenberg (32 ms) and that observed for the present study (35 ms) is very typical for the task (Lu and Proctor, 1995) and this unanticipated language-group difference cannot be attributed to either an unusually weak or unusually strong instantiation of the Simon task. It would be risky to assume that our Simon data reflects a monolingual advantage in general inhibitory control because the same set of studies show no language-group differences with respect to the magnitude of the flanker interference-effect or in switching costs. This inconsistency across measures will be discussed further in the discussion of convergent validity.

The global RT measure for the Simon task also showed a statistically-significant monolingual advantage. However, this advantage appears to be spurious, or at least inconsistent with the combined analysis of Paap and Greenberg's Studies 1–3 that showed a very small and non-significant monolingual advantage (5 ms) in global RT. As global RT is a very impure measure of monitoring, it is also informative that the purer measure of Simon mixing costs actually favors bilinguals, although that advantage is far from significant. Likewise the significant monolingual advantage in antisaccade RT was not significant in Study 1 of Paap and Greenberg (2013), nor were there any significant language-group differences in the present study for either of the two measures based on antisaccade accuracy or in the measure of antisaccade RT costs. In summary, the only consistent difference observed across the four fairly large n studies conducted in our laboratory is a monolingual advantage in the Simon interference effect.

## Results for convergent validity of EF tasks

### Switching

Switching costs derived from the color-shape switching task were not correlated with the shifting costs (sequential dependencies) derived from the mixed blocks of the ANT task, *r* = +0.04, *p* = 0.664. Although some researchers (e.g., Bialystok and Barac, 2012) have assumed that task switching and trial-to-trial shifts in congruency are more-or-less equivalent, the dissociation between the two measures cautions that they should not be treated as the same function.

In the Miyake and Friedman studies the measures from the three switching tasks were significantly correlated, strongly loaded on the shifting factor, and the shifting factor was separable from the updating function. An investigation of the implicit association test by Klauer et al. (2010) used a similar set of switching tasks found significant cross-task correlations for two of the three tasks, both of which strongly loaded on the switching factor, and the switching factor was related to (but separable from) both the inhibition and WMC factors. Overall, switching tasks appear to enjoy good levels of convergent validity.

### Monitoring

Table 6 shows the cross-task correlations for the five assumed measures of the monitoring component. Global RT in the ANT task is significantly correlated with global RT in the Simon task (*r* = +0.60), but a host of non-executive processes may be contributing to the association. This possibility is reinforced by observing that the mixing costs for the Simon and ANT tasks yield a negative and non-significant correlation (*r* = −0.09). Because mixing costs in the color-shape switching task are also assumed to reflect the monitoring component of EF, this measure can also be correlated with the mixing costs obtained in the Simon and ANT tasks: the correlation between mixing in the switching task and mixing in the ANT task is near zero (*r* = +0.02), whereas the correlation between mixing in the switching task and mixing in the Simon task is significant (*r* = −0.22), but inexplicably negative^9^. The present results reinforce the need for additional work in operationally defining constructs like monitoring, coordination, or mental flexibility and establishing their validity as measures of EF. The measures currently used show no convergent validity.

**Table 6:** **Correlations between assumed measures of the monitoring component of EF**.

**Measures of monitoring**		**Measures of monitoring**				
		**A**	**B**	**C**	**D**	**E**
A. Flanker mixing costs	r	+1.00	+0.162	−0.091	+0.009	+0.025
	p		0.087	0.352	0.927	0.796
B. Flanker global RT	r		+1.00	−0.085	+0.597	+0.378
	p			0.384	**<0.001**	**<0.001**
C. Simon mixing costs	r			+1.00	+0.225	−0.224
	p				**0.019**	**0.021**
D. Simon global RT	r				+1.00	+0.233
	p					**0.014**
E. Mixing cost (switch task)	r					+1.00
	p					

*r, Pearson correlation; p, exact probability. Cross-task correlations significant with p < 0.05 are shown in bold. Operational definitions of the 5 measures appear in Table 3*.

### Inhibitory control component

Table 7 shows the cross-task correlations for the seven assumed measures of inhibitory control. As discussed earlier, measures of inhibitory control derived from the flanker, Simon, and Stroop tasks have often shown low levels of convergent validity making it highly likely that the conflict resolution mechanisms employed are task specific rather than recruiting general-purpose inhibitory control. In the present study the magnitude of the Simon effect does not significantly correlate with the magnitude of the flanker effect (*r* = +0.14). Measures derived from the antisaccade task produced significant cross-task correlations in both the Miyake and Friedman studies and the Unsworth studies, but in the present study not one of the four antisaccade measures yielded significant cross-task correlations with either the flanker or Simon effect, although the correlation between the Simon effect and antisaccade RT came close, *r* = +0.17, *p* = 0.07.

**Table 7:** **Correlations between assumed measures of the inhibitory control component of EF**.

**Measure of inhibitory control**		**Measure of inhibitory control**						
		**A**	**B**	**C**	**D**	**E**	**F**	**G**
A. Flanker effect	r	+1.00	+0.140	+0.041	+0.001	+0.026	+0.071	+0.021
	p		0.142	0.679	0.988	0.791	0.472	0.826
B. Simon effect	r		+1.00	+0.175	+0.066	+0.025	+0.069	+0.168
	p			0.072	0.497	0.802	0.478	0.076
C. Antisaccade RT	r			+1.00	+0.446	0.312	+0.397	+0.397
	p				**<0.001**	**0.001**	**<0.001**	**<0.001**
D. Antisaccade PC	r				+1.00	+0.232	0.777	+0.072
	p					**0.015**	**<0.001**	0.461
E. Antisaccade RT costs	r					+1.00	+0.285	+0.303
	p						**0.003**	**0.002**
F. Antisaccade PC costs	r						+1.00	+0.072
	p							0.461
G. Switch costs (switch task)	r							+1.00
	p							

*r, Pearson correlation; p, exact probability. Cross-task correlations significant with p < 0.05 are shown in bold. Operational definitions of the 7 measures appear in Table 3*.

Perhaps the most informative association revealed in the present study is that both antisaccade RT (*r* = +0.40, *p* < 0.001) and antisaccade RT costs (*r* = +0.30, *p* = 0.002) significantly correlated with the *switching costs* derived from the switching task. This correlation supports the view that switching involves not only the instantiation of a new goal with a new set of relevant information, but also the suppression of information relevant to the previous goal. It is also consistent with Miyake and Friedman's report that the latent variables for switching and inhibition were highly correlated.

## Discussion

### Consequences of the lack of convergent validity

As described above dependent variables that have been assumed to measure either the inhibitory control or monitoring components of EF show weak associations at best. How dire the implications are is a matter of perspective and for Salthouse (2010) they are grim. To demonstrate his concern Salthouse had 265 participants (in three age ranges) complete two versions of the flanker task that were identical except for the materials. In the arrow version an incongruent trial looked like this: << > <<; whereas the letter version an incongruent trial look like this: GG H GG. Twenty practice trials were followed by 100 experimental trials consisting of 50 randomly ordered congruent and incongruent trials. Both versions yielded highly significant flanker interference effects, but the correlation between the two interference effects is surprisingly low, *r* = +0.03, and non-significant^10^.

Regardless of what the flanker task is assumed to measure it may not be useful in individual differences comparisons. If the conflict present on the incongruent trials was resolved by employing an inhibitory mechanism not required on the congruent trials, then the subtraction should capture individual differences in that inhibitory mechanism and the cross-task (e.g., letter vs. arrow) measures should correlate. That they do not, is more consistent with a conceptual framework of the flanker task that assumes that the same operations take place in both conditions, but that those operations take longer and are more error prone on incongruent trials. Salthouse arrives at these disconcerting thoughts: “These results suggest that the flanker task may be sensitive to conflict, but that at least in the current versions of the task, there are little or no systematic individual differences in the magnitude of the behavioral manifestations of this conflict. This suggestion merits additional research because it raises questions about the wide-spread practice of using performance in flanker tasks to assess individual differences in aspects of executive functioning…” (p. 59).

Pragmatically speaking it is worthwhile to mention additional reports of significant cross-task correlations within components of EF. For 8–15 year olds the NIH Toolbox version of the flanker task (including both a simple fish block and a more difficult arrow block) did show convergent validity with the D-KEFs Inhibition raw scores (viz., the incongruent condition of a Stroop color-word interference task), *r*_(81)_ = 0.34, *p* < 0.002 (Zelazo et al., 2013). Each block consisted of 16 congruent and 9 incongruent trials presented in pseudorandom order. The toolbox flanker task does not include the various types of precues required to do an ANT analysis.

Although the cross-task correlations and factor loadings in Klauer et al. are smaller for their Inhibition factor compared to the Switching and WM factors, their four interference tasks display cross-task correlations somewhat stronger than those obtained by Miyake and Friedman and much more promising than those obtained in the present study and many previous studies. More specifically, the factor loadings were 0.61 for the antisaccade task, 0.46 for the Simon, 0.35 for the Stroop, and 0.24 for the flanker task. Given their popularity in the bilingualism literature, one notes that the zero-order correlation between the Simon and flanker effects was significant, *r* = 0.21, *p* < 0.05. The flanker task was the same as that used by Friedman and Miyake (2004), did not include the ANT version precues, but had increased working-memory demands as there were two letters, rather than just one, assigned to each response. The targets in the Simon tasks were arrows pointing left and right and there were 72 congruent and 72 incongruent trials.

Another recent “success” at securing significant convergent validity in interference control uses tasks described in Wostmann et al. (2013). For present purposes the cross-task correlations of interest were those between Simon interference and flanker interference (*r* = 0.24, *p* < 0.01), Simon and Stroop (*r* = 0.28, *p* < 0.01), and Stroop and flanker (*r* = 0.11, *p* = 0.001) for a group of 534 adult participants (personal communication from Ulrich Ettinger, Feb. 5, 2014). The targets used in the Simon task were arrows pointing left or right, but there were 160 congruent trials compared to only 60 incongruent. This produced an unusually large Simon effect of 71 ms. The flanker task was the arrow version and included 40 trials each of congruent, incongruent, and neutral trials.

This brief review of cases where interference measures significantly converged suggests that Simon tasks with arrow targets (and perhaps with less frequent incongruent trials) may produce larger Simon effects and more convergence than other variants. The tea leaves are even more difficult to read for the flanker task, but significance convergence seems to occur when the contrast between congruent and incongruent trials is not perturbated by the varying precue conditions needed to implement the ANT version of the flanker task. It may also be the case that making the flanker task more difficult by having multiple targets assigned to each response may amplify the cross-task correlations. Another aspect of the overall pattern is that significant cross-task correlations on interference measures are more often obtained when the n's are very large. Thus taking a broad view, that glosses over differences in how tasks are instantiated, one might conclude that the interference effects obtained in the antisaccade, flanker, Simon, and Stroop tasks do show low-levels of convergent validity that will usually require large n's to detect.

### Cognitive abilities do not adhere to a hierarchical structure

Relations between cognitive variables are often organized based on the pattern of correlations. One useful organization is a hierarchy with observed variables at the lowest level, various cognitive abilities at intermediate levels, and a general fluid intelligence (gF) factor at the highest level. Cattell (1971) originally defined gF as the ability to discriminate relations and in contemporary usage it is often conceptualized as the ability to reason, solve novel problems, and adapt to new situations (Salthouse et al., 2008). Because EF components like monitoring, updating, switching, and inhibiting logically serve successful reasoning, problem solving, and adapting; EF should be related to gF. On the other hand, high quality reasoning seems to require more than the sum of the parts of EF. This is, for example, consistent with the assumption that the reasoning about relationships required by the Ravens test leads to its characterization as a measure of gF and not EF. Putting these hierarchically-driven assumptions together, measures of the same component of EF (e.g., two measures of inhibitory control) should strongly correlate, measures of EF that cross components should moderately correlate (e.g., measures of inhibitory control and switching) and measures of EF and gF should have the smallest correlation. Unfortunately, the evidence does not support this hierarchical structure. As already discussed tasks designed to measure the same component of EF are weakly associated at best. The next sections shows that EF and gF are highly related and difficult to separate.

### Consequences of the lack of discriminant validity

Salthouse also showed that multiple measures of gF were strongly related to several measures of EF (Salthouse et al., 2003, 2008). For example, in Salthouse et al. (2008) the three updating variables were highly related to gF; with the running memory requirement of having to continuously update the most recently presented four items as strongly related to the gF factor as the gF variables were to each other.

Salthouse et al. (2003) similarly examined the convergent and divergent validity of several “neuropsychological variables” including the Wisconsin Card Sorting Task (WCST), a three-ring tower puzzle, a variant of the trail-making test, and verbal fluency to letter probes. When these neuropsychological variables were forced to load on the same latent variable (labeled EF) they showed moderate convergent validity with a mean loading of 0.54. Salthouse often uses a core set of “reference” factors (viz., gF, memory, processing speed, and vocabulary) that have demonstrated convergent and divergent validity. When the neuropsychological variables are allowed to load on any of the constructs all of the “neuropsychological variables” that previously loaded on EF became non-significant as each variable is more strongly associated with one or more members of the core constructs. For example, WCST loads primarily on gF (0.42), whereas verbal fluency loads on both Speed (0.41) and Vocabulary (0.36). One can certainly quibble about what processes, abilities, or functions should be treated as core, but this type of analysis underscores the importance of examining whether measures used to test for language group differences in EF diverge from other cognitive abilities.

If gF encompasses a broad spectrum of controlled processing, then investigators working from different research traditions may be giving different names to the same dimension of individual differences. “Whether the dimension is labeled gF, working memory, or executive processing, or some form of cognitive control may reflect the research tradition of the investigator more than any fundamental differences among the concepts because it appears that individuals would be ordered in nearly the same way with variables from each of these perspectives” (Salthouse, 2010, p. 484).

The hierarchical structure perspective also invites consideration of the role of EF as a theoretical construct in laboratory tasks compared to its role in sustaining self-control in everyday life. Duckworth and Kern (2011) point out the extraordinary diversity of self-control as operationalized in various research studies: refrain from pushing a button when a stop signal occurs, choose between one marshmallow now or two later, your self-rated longing for excitement, or your predilection for making up your mind quickly. In a very large meta-analysis of 282 samples and *N* = 33,564 participants they conclude that self-control is a coherent and multidimensional construct. For the present purpose of examining the convergent validity between measures of EF it interesting to discover that cross-task correlations across different types of self control were strongest for informant-report questionnaires and weakest for laboratory-based EF tasks; the very set relied upon to test for bilingual advantages in EF.

## Conclusions

### Are these 13 indices measuring EF ability?

The cross task correlations between indices assumed to measure the same component of EF are not encouraging. For inhibitory control the only measures that converged were antisaccade RT and switching costs. The complete absence of convergence for the flanker and Simon effects in the present data and in several previous studies suggests that researchers should stop using these tasks, or replace them with versions that have demonstrated convergent validity. Likewise there is no convergent validity between difference-score measures assumed to reflect monitoring ability. The significant cross-task correlations in global RT are contaminated by individual variation in basic perceptual and motor processing. Although switching costs in the switching task did not correlate with shifting costs in the flanker task, the present design did not include multiple measures of switching between tasks. Given the significant factor loadings reported by Miyake and Friedman (2012) and Klauer et al. (2010) between three different switching tasks and the latent variable for Switching these switching tasks appear to provide an adequate test for this component of EF.

### Implications for the study of bilingual advantages

The low (or zero) levels of convergent validity compel caution in the interpretation of any bilingual advantage in performance, even if the n is large and the groups are exquisitely matched with respect to important demographic characteristics. The low levels of convergent validity imply that these measures are reflecting task-specific mechanisms rather than the efficacy of general functions. The lack of divergent validity between EF and gF constructs is yet another warning that we may not be measuring what we think or hope to measure. Ending with a mea culpa regarding the tasks used in the present study, the field needs to consistently identify and use measures that show better convergent validity and that diverge from cognitive abilities that are assumed to be unaffected by managing two languages.

### Conflict of interest statement

The authors declare that the research was conducted in the absence of any commercial or financial relationships that could be construed as a potential conflict of interest.

## Footnotes

^1^See Unsworth et al. (2014) for an example that used variance partitioning and cluster analysis to identify subgroups that differ in terms of attention control, secondary memory, and capacity.

^2^The precues that define the ANT task enable the measure of alerting and orienting networks. The difference between congruent and incongruent trials is referred to as “attentional control” by Fan et al. but we will refer to this difference score as the flanker effect to be consistent with the name for the same difference score in highly similar “flanker” tasks that do not use the spatial or temporal precues.

^3^Miyake and Friedman refer to this component as “shifting,” but the term “switching” is used more often in the literature on bilingualism.

^4^In addition to the standard Stroop where colors were incongruently paired with words, arrows were incongruently paired with words, small-component digits were incongruently paired with the larger digit they formed, and a count of the number of digits in a row was incongruently paired with the value of the repeated digit.

^5^Our use of the term inhibitory control ignores the distinction between interference control (suppression of interference due to resource or stimulus competition) and response inhibition (suppression of prepotent responses). The task-impurity problem makes it very difficult to isolate the different interference-related processes and Friedman and Miyake (2004) have shown that separate latent variables for interference control (e.g., the flanker effect in their study) and response inhibition (e.g., antisaccade and Stroop effects) are highly correlated (0.68). In their other studies, where both interference control and response inhibition tasks are forced to load on one “inhibiting” factor, the factor loadings are only moderate in size.

^6^In recent studies (Unsworth et al., 2014) WM is further distinguished between the manifest measures of storage capacity (WM-S) and the measures of processing speed on the “operations” task (WM-P).

^7^It is generally agreed that switching costs may reflect not only the ability to switch mental sets, but also the ability to replace or inhibit the rule active on the previous trial.

^8^The typical Simon effect is much smaller than the typical flanker, Stroop, or task switching effect and, consequently, the conflict may not rise to the same level of conscious awareness. The role this may play in amount of processing resources allocated to conflict monitoring would be an interesting topic for future research.

^9^In Paap and Greenberg (2013) the correlations between mixing-costs in the Simon and switching tasks were *r* = 0.00 (Study 2) and *r* = −0.04 (Study 2).

^10^As a general practice, Salthouse recommends that instead of isolating by subtraction one should use the simple condition (e.g., congruent trials) as a covariate in analyses of the complex condition (e.g., incongruent trials). The method removes the linear effects of the simple condition and all subsequent analyses can be conducted on the residual of the complex condition. For the flanker data, this method also yields a non-significant correlation between the letter and arrow tasks, *r* = +0.04.
