# Supplementary material for: Bilingual advantages in executive functioning: problems in convergent validity, discriminant validity, and the identification of the theoretical constructs
Source: Front Psychol. 2014 Sep 9;5:962. doi: 10.3389/fpsyg.2014.00962 (PMC4158994; doi:10.3389/fpsyg.2014.00962)
Supplement: Supplementary file 1 [file Presentation1.PDF]

APPENDIX

Prompts and response categories used to solicit background information on video gaming frequency, frequency of multitasking, attitude toward multitasking environments, and ability at team sports.

How often do you play computer games that require you to attend to many things at the same time and make fast appropriate responses?

1. never
2. a few times a year
3. about once a month
4. several times a week
5. every day, but usually for less than an hour
6. every day, and usually for more than an hour

How often do you listen to music or other audio when you are walking, running, or exercising?

1. never
2. a few times a year
3. about once a month
4. several times a week
5. every day, but usually for less than an hour
6. every day, and usually for more than an hour

How often do you listen to music or other audio when you are doing homework, reading, or other cognitive tasks?

1. never
2. a few times a year
3. about once a month
4. several times a week
5. every day, but usually for less than an hour
6. every day, and usually for more than an hour

How often do you talk or text on a cell phone when you are doing homework, reading, or other cognitive tasks?

1. never
2. a few times a year
3. about once a month
4. several times a week
5. every day, but usually for less than an hour
6. every day, and usually for more than an hour

Compared to a typical college student, how often do you multitask, that is, engage in two or more tasks at the same time?
1. far less than average
2. somewhat less than average
3. average
4. somewhat more than average
5. far more than average

Team sports often involve dividing your attention between a ball, a goal, your opponents, and your teammates. Do you excel at these sports?
1. not at all
2. I'm ok
3. I'm average
3. I'm better than average
4. I'm significantly better than average
5. I'm much better than average

How do you feel when you need to focus on an important task but there are lots of things going on that could be distracting? I find these situations:
1. very frustrating and my performance is usually not as good as it could be.
2. somewhat frustrating and my performance is sometimes not as good as it could be.
3. neither frustrating nor stimulating.
4. somewhat stimulating and it sometimes actually improves my performance.
5. very stimulating at it usually helps me to perform better.
